# Rapid Analysis of Inorganic Species in Herbaceous Materials Using Laser-Induced Breakdown Spectroscopy

**DOI:** 10.1089/ind.2015.0019

**Published:** 2015-12-01

**Authors:** Tyler L. Westover, Rachel M. Emerson

**Affiliations:** Energy Sciences Laboratory, Biofuels and Renewable Energy Technologies Department, Idaho National Laboratory, Idaho Falls, ID

## Abstract

Inorganic compounds in biomass, often referred to as ash, are known to be problematic in the thermochemical conversion of biomass to bio-oil or syngas and, ultimately, hydrocarbon fuels because they negatively influence reaction pathways, contribute to fouling and corrosion, poison catalysts, and impact waste streams. The most common ash-analysis methods, such as inductively coupled plasma-optical emission spectrometry/mass spectrometry (ICP-OES/MS), require considerable time and expensive reagents. Laser-induced breakdown spectroscopy (LIBS) is emerging as a technique for rapid analysis of the inorganic constituents in a wide range of biomass materials. This study compares analytical results using LIBS data to results obtained from three separate ICP-OES/MS methods for 12 samples, including six standard reference materials. Analyzed elements include aluminum, calcium, iron, magnesium, manganese, phosphorus, potassium, sodium, and silicon, and results show that concentrations can be measured with an uncertainty of approximately 100 parts per million using univariate calibration models and relatively few calibration samples. These results indicate that the accuracy of LIBS is comparable to that of ICP-OES methods and indicate that some acid-digestion methods for ICP-OES may not be reliable for Na and Al. These results also demonstrate that germanium can be used as an internal standard to improve the reliability and accuracy of measuring many elements of interest, and that LIBS can be used for rapid determination of total ash in biomass samples. Key benefits of LIBS include little sample preparation, no reagent consumption, and the generation of meaningful analytical data instantaneously.

## Introduction

Inorganic constituents in biomass, often referred to as ash, contribute to several problems in conversion processes, such as fouling of reactor surfaces and effects on reaction pathways that reduce yields and increase waste streams. The total ash content and constituents in biomass are known to vary over a wide range and are influenced by plant type, growing conditions, harvesting methods, and handling operations. The current methods for determining ash content in biomass include inductively coupled plasma-optical emission spectrometry (ICP-OES), inductively coupled plasma-mass spectrometry (ICP-MS), and flame atomic absorption spectrometry (FAAS).^1–4^ These methods generally require significant sample preparation, consuming both reagents and time. More rapid and economical means of ash analysis would not only reduce analytical costs but would also facilitate in-line blending of biomass materials with different ash compositions to achieve ash specifications for specific conversion processes.

Laser-induced breakdown spectroscopy (LIBS) is currently being investigated as a method for rapid screening and determination of the mineral concentrations of a wide variety of biomass. LIBS is low cost, requires little sample preparation, and can provide immediate results. It is a potential cost-efficient alternative for rapid screening of biomass feedstocks. In LIBS, a small sample of material is ablated with a high-energy pulsed laser, producing a high-temperature plasma above the sample surface. The plasma contains atoms and ions from the sample that emit at frequencies according to atomic energy states and transition probabilities. Using a spectrometer to measure spectral peaks emitted from the plasma makes it possible to identify practically all elements—organic and inorganic—that are present in the sample in concentrations ranging from parts per billion to several percent. Furthermore, LIBS spectra can be calibrated using reference materials with known elemental analysis, allowing for rapid quantitative measurements of elemental concentrations. Important parameters in the optimization of the LIBS signal for particular elemental and ionic peaks include the period of time after the laser fires before the shutter of the spectrometer opens (gate delay); the time that the spectrometer acquires emitted light (gate width); and the spot size, power, pulse time, and pulse frequency of the laser.

Recent years have witnessed considerable activity employing LIBS to characterize numerous inorganic materials; however, relatively few reports involve biological materials.^5–9^ Sun et al. used National Institute of Standards and Technology (NIST)-certified standard reference materials (SRMs) to construct LIBS calibration curves, which were employed with LIBS analysis to determine the concentration of calcium (Ca), magnesium (Mg), phosphorus (P), iron (Fe), copper (Cu), manganese (Mn), zinc (Zn), and aluminum (Al) in plant leaves.^10^ It was found that a gate delay of 1 μs and an integration time of 10 μs provided the optimum signal-to-noise ratio, and the coefficient of variation for measurements was approximately 8–15%. Cho et al. mixed starch powder with NASBA International Evaluation Services (NIES) SRMs to improve sample rigidity for optimal plasma formation and found detection limits of 0.4 and 3 μg/g for magnesium (Mg) and potassium (K), respectively.^11^ Gornushkin et al. investigated the influence of the sample matrix on Mg signals in powdered samples and proposed a surface-density normalization method to compensate for quantitatively problematic matrix effects.^12^ In detailed studies, Nunes et al. optimized LIBS parameters and validated the LIBS approach for the determination of macro- and micronutrients in pelletized sugar cane leaves.^13^ A total of 41 laboratory reference samples were used to construct calibration curves using different techniques. Repeatability of measurements obtained by univariate and multivariate calibrations ranged from 1.3–29% and 0.7–15%, respectively, demonstrating that LIBS can be a powerful tool for analysis of pelletized plant materials. Additional studies have shown that calibration curves can be constructed for SRMs using both univariate and multivariate approaches.^14,15^ Expensive femtosecond lasers have also been used to analyze inorganic species in leaves and roots with high spatial resolution and sensitivity.^16,17^

The principal challenge in using LIBS as a potential feedstock screening technique is due to poor correlation of certain measured LIBS intensity values with analytical concentration values in several elements.^5^ These poor correlations manifest as apparent outliers or spread in the data outside of tolerance limits. There are several potential causes for the presence of these discrepancies, including the inability of LIBS to measure mineral composition of widely varying materials with a single calibration curve (often referred to as matrix effects), interactions between elements in the plasma plume that influence LIBS intensity data, and possible errant analytical concentration data used to build the calibration curve. The cause of these correlation effects need to be determined and mitigated before LIBS can be employed reliably to analyze inorganic constituents (ash) in biomass feedstock materials.

The purpose of the present study is to demonstrate that LIBS offers an approach for rapid analysis of ash species that are common in biomass materials using simple techniques and relatively inexpensive equipment. This study also provides possible causes and solutions for the difference in matrix effects that have been shown to reduce calibration accuracies. Twelve different biomass samples of widely varied ash composition were used to build LIBS calibration curves. Six of the materials were SRMs from NIST. Six other biomass materials were also chosen, and the mineral concentrations of these additional biomass samples were determined by independent ICP-OES/MS analysis. LIBS calibration curves were developed by comparing LIBS results from element concentration values obtained from NIST and independent ICP-OES/MS methods.

This work is unique in several respects. First, it contains the first published report of employing germanium (Ge) powder in biomass as an internal standard, which is shown to improve calibration fits and reduce measurement uncertainty. Second, elemental ash analysis of 12 samples, including six SRMs, using three ICP-OES/MS methods are compared with certified concentration values where available, and the strengths and weaknesses of the different analytical methods are discussed. Third, this work demonstrates that LIBS can be used for rapid analysis of total ash in biomass samples with very good accuracy if the proper calibration is applied for each sample type and ash species. Understanding the limitations associated with the LIBS calibrations for different sample types and ash species is crucial to obtaining accurate elemental ash analysis using LIBS instruments, especially when budget or time constraints limit the number of reference samples that can be used to develop instrument calibration models.

## Materials and Methods

### Samples

Samples from 12 different types of biomass were used for the evaluation of LIBS. Six of the samples were NIST SRMs, which were ground to −75 microns and have certified concentrations of inorganics. The following six SRMs were used for calibration: NIST 1515 apple leaves (ApLe), NIST 1547 peach leaves (PeLe), NIST 1570a spinach leaves (SpLe), NIST 1573a tomato leaves (ToLe), NIST 1575a pine needles (PiNe), and NIST 1567a wheat flour (WhFl). The other six samples, which are common materials of interest as potential feedstocks for biofuels production, include: corn stover (CnSt), miscanthus (Misc), reed canary grass, (ReCG), sorgum (Sorg), switchgrass (SwGr), and wheat straw (WhSt). The samples were prepared by grinding them to −80 μm using a knife mill (ZM200, Retsch Technology GmbH, Haan, Germany). Pellets were made for LIBS analysis from the ground material by placing approximately 1 g of mixed sample powder in a 0.5-in diameter pellet die and applying approximately 700 MPa pressure for 1 min. For an internal standard, Ge powder (particle size less than 50 μm) was added to the biomass at known (2–4%) concentrations after the biomass was ground. The biomass with the Ge powder was thoroughly mixed following the method published by NIST on the SRM certificates and then pelletized following the procedure described above.

### ICP-OES/MS

After being ground to −80 μm, three methods were employed to prepare the samples for ICP-OES/MS. The first method, referred to as the HF-acid method, was applied at Idaho National Laboratory (Idaho Falls, ID) by digesting the sample powders in closed beakers using a microwave-assisted process in hydrofluoric (HF) acid. The second and third methods were both employed at Huffman Laboratories (Golden, CO). In the second method, referred to as the 2-acid method, the samples were digested in duplicate with nitric (HNO_3_) and refluxing perchloric (HClO_4_) acids to fully oxidize all organic material present. In the third method, referred to as the LMF-method, the samples were first staged by ashing at 750°C for 8 h and then subjected to a lithium metaborate fusion process.

### LIBS

A modified RT-100 (Applied Spectra, Fremont, CA) was used to perform the LIBS measurements. In this instrument, laser excitation was provided by a pulsed neodymium-doped yttrium aluminium garnet (Nd:YAG) laser with a wavelength of 1,064 nm and a 7 ns pulse in air. The laser was capable of a maximum power of 90 mJ per pulse and was operated at 80% of maximum. The laser focusing lens had a focal length of 15 mm. A 5-channel spectrometer system with approximately 1.5 nm resolution was used with a charge couple device (CCD). A schematic of the system is shown in *Fig. S1* (Supplementary Data are available online at www.liebertpub.com/ind). LIBS spectra were collected at 20 different test sites on each side of each pellet (40 sites total) and were accumulated at each site from 20 consecutive laser pulses for improved precision. During the tests, the gate delay time was set at 1 or 3 μs, the gate width was set at 1 ms, and the lens-to-sample distance was adjusted to assure high signal-to-noise ratio and low standard deviation of measurements. Multiple replicates of each sample were tested to ensure repeatability and precision of results.

### Data Analysis

Because many elements exhibit signature peaks at multiple wavelengths, the entire wavelength range from 200–1,000 nm was analyzed to identify promising peaks for each element of interest. Each peak of interest in the collected spectra was identified based on NIST and other spectroscopic databases. In some cases, peaks from different elements can overlap in the same wavelength region, causing interference and making it difficult to determine the concentrations of particular elements using only a single peak for each element. To avoid such difficulties and to ensure that the LIBS calibration models were as robust as possible, multiple peaks were identified for each element. The spectral peaks for each element were evaluated for use in the calibration curve according to signal-to-noise ratios, strength of spectral peaks, and interferences from adjacent peaks. Reported spectral peaks were chosen based on optimized linear correlations between spectral peak intensity values and measured concentration values.

Spectral data were imported into Microsoft Excel for data processing. A baseline correction was determined by fitting a straight line through several data points on each side of each spectral peak of interest, and care was taken to ensure that the intervals selected for the baseline determination did not contain any visible peaks. After the baseline correction was determined, it was subtracted from the peak of interest. Peak intensities were measured by determining the area under the spectral peak after baseline correction. For the SRMs, measured LIBS peak intensities were plotted as a function of concentration certified by NIST, and calibration curves were calculated following the development described by Draper and Smith.^18^ The magnitudes of the fit residuals suggested that the errors between the best fit lines and measured LIBS intensities increased somewhat with LIBS intensity but did not follow Poisson statistics, in which the uncertainty in measured signal intensity is proportional to its own square root.^19^ Consequently, it was assumed that uncertainties in the LIBS measurements were approximately constant (i.e., independent of the magnitude of the measured LIBS intensity) and followed a normal distribution, allowing estimation of 95% confidence intervals for measurements.

It is important to note that multivariate calibration methods based upon large numbers of similar reference materials typically yield more reliable results with better precision than univariate calibration methods, as demonstrated by Nunes et al.^13^ However, large numbers of samples must be employed to develop multivariate models to ensure that such models are not dependent upon experimental noise, which decreases their reliability. As discussed below, the number of samples in the present study is not sufficient to justify developing robust multivariate models.

In addition to building calibration curves based on the measured intensities of all identified LIBS peaks, the measured intensities were also normalized using the measured intensities of the hydrogen peak at 656 nm, the carbon peaks at 248.9 nm and 909.5 nm, as well as Ge peaks at 265 nm and 304 nm for samples that had been doped with Ge. As these elements have consistent concentrations for the samples in this study, this normalization technique helped to account for instrument drift and other external factors of the analysis. Normalization by the carbon peak at 248 nm (C I 248.9 nm) and the Ge peak at 265.1 nm (Ge I 265.1 nm) exhibited the best fit performance, so only those results are discussed below.

## Results

**Fig. 1.**
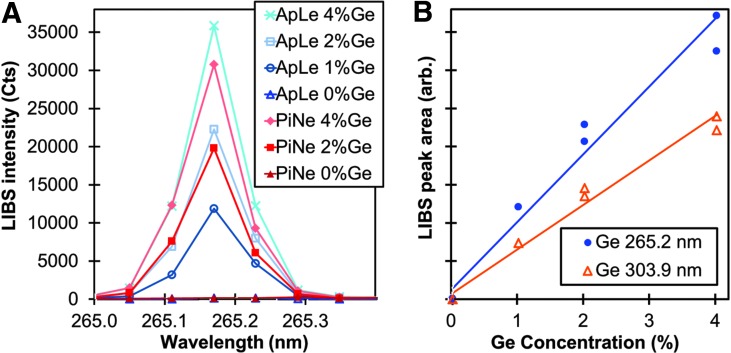
LIBS data from NIST 1515 apple leaves and NIST 1575 pine needles mixed with 0–4% Ge powder. **(A)** LIB spectra near the Ge 265 nm peak; **(B)** Calculated areas under the Ge 265.2 nm peak in **(A)** and the Ge 303.9 nm peak as functions of known Ge concentration; GD = 2.0 μs for all spectra. Color images available online at www.liebertpub.com/ind

*Figure 1A* contains LIBS spectral data of the Ge I 265 nm peak from NIST 1515 ApLe and NIST 1575a PiNe mixed with 0–4% Ge powder by weight. Importantly, the area under each curve increases with Ge concentration, and the fact that the peaks obtained from both the apple leaves and the pine needles are approximately the same size for similar levels of Ge concentration indicates that both of these materials respond to the laser excitation in approximately the same way (i.e., both samples exhibit nearly the same matrix effects for this spectral range). The areas under the LIBS peaks in *Fig. 1A* are plotted in *Fig. 1B* as functions of Ge concentration. The results from a similar calculation for the areas under the Ge I 303 nm peak are also shown and demonstrate that the relationship between the LIBS response and Ge concentration is approximately linear, especially for Ge concentrations less than 2%. These results indicate that Ge is a suitable internal standard, and importantly, it is not present in most native biofuel feedstocks.

*Table 1* summarizes the LIBS peaks that were found most helpful for obtaining calibration curves to analyze inorganic species in the samples tested in this work. Results are presented for the optimal gate delay (GD) and spectra-normalization method (none, normalization by the C I 248 nm peak, or normalization by the Ge I 265 nm peak) for each elemental peak of interest. Separate linear fits were created for the NIST SRMs and the non-SRM samples, because in many instances the non-SRM samples exhibited calibration trends that were different from those of the NIST SRMs for many of the peaks. The calibration curves that were developed for the various elements of interest for the SRMs and the non-SRM samples are described below. Note that the coefficient of determination (*R*^2^) for nearly all of the fits is greater than 0.95, which is remarkable considering the mild disagreement in the calibration data, as discussed below.

**Table 1.** LIBS Spectral Peaks Found Most Useful for Building LIBS-Calibration Curves. The Optimal GD for Each Peak and the LIBS-Calibration Curve Parameters, Including Curve Sensitivity (Slope, *b*), Coefficient of Determination (*R*^2^) and 95% Prediction Uncertainty (Standard Error, *U*_95%_)

		NIST SRMs					NON-NIST SRM SAMPLES				
SPECIES	PEAK POSITION (nm)	GD (μs)	NORMALIZATION	*b* (cts/ppm)	*R*^2^	*U*_95%_ (ppm)	GD (μs)	NORMALIZATION	*b* (cts/ppm)	*R*^2^	*U*_95%_ (ppm)
Al I	394.5	3	C248	0.57	0.97	45	1	None	0.66	0.98	51
Al I	308.3	3	None	4.93	0.98	37	3	None	3.22	0.98	49
Ca II	370.6	3	C248	0.09	1.00	809	1	C248	0.05	0.96	93
Ca II	849.8	3	C248	0.31	1.00	691	1	Ge265	0.07	0.90	144
Ca II	315.9	3	C248	0.14	0.99	1098	1	C248	0.08	0.90	150
Fe I	438.3	1	Ge265	1.33	1.00	8	1	None	2.6	0.99	44
Fe II	259.9	1	None	6.19	0.98	18	1	None	16	0.96	83
Fe II	261.2	1	None	0.45	0.97	24	1	None	0.90	0.99	46
K I	766.5	3	None	7.9	0.98	1488	1	None	8.0	0.96	912
K I	693.9	3	None	0.28	0.92	3034	3	None	0.27	0.93	1088
K I	404.5	1	Ge265	0.07	0.94	2525	3	None	0.06	0.76	1954
Mg I	518.3	3	None	0.29	0.99	277	1	Ge265	0.17	0.99	83
Mg II	278.5	3	None	0.01	0.99	335	1	Ge265	0.03	0.91	234
Mg I	309.1	3	None	14.8	1.00	233	1	Ge265	7.9	1.00	51
Mn II	257.6	1	Ge265	2.94	1.00	7	1	Ge265	7.9	0.80	9
Mn I	403.2	1	Ge265	7.9	1.00	7	1	Ge265	17	0.74	10
Na I	589	1	Ge265	1198	0.91	13	1	None	392	0.97	60
Na I	819.5	1	C248	16	0.81	17	3	None	2.25	0.96	68
P I	213.6	1	C248	0.03	0.99	170	1	Ge265	0.03	0.92	162
P I	255.3	1	C248	0.03	0.96	287	1	Ge265	0.02	0.94	139
Si I	251.6	1	None	0.59	0.75	604	3	C248	0.29	0.98	1702
Si I	243.6	1	None	0.02	0.80	544	3	C248	0.01	0.99	1268
Si I	288.2	1	None	0.21	0.87	441	3	C248	0.12	0.98	1368

**Fig. 2.**
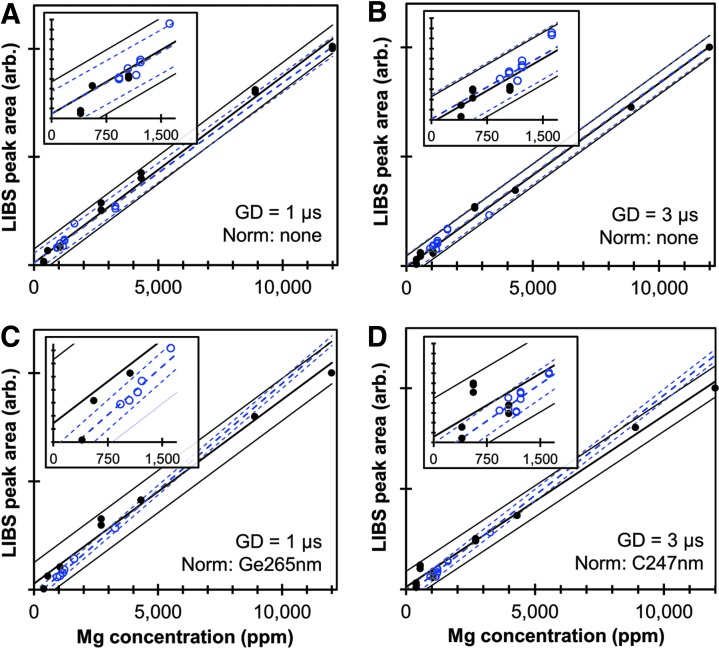
LIBS calibration data for magnesium using the Mg II 278.5 nm peak. The area under the peak is plotted as a function of estimated Mg concentration for all SRMs (solid dots) and non-SRM samples (hollow circles). The thick solid and dashed lines represent the least-squares fit for the SRM and non-SRM datasets, respectively, while the thin solid and dashed lines represent the 95% confidence intervals for the SRM and non-SRM fits. **(A)** GD 1 μs; **(B)** GD 3 μs; **(C)** normalized by the C peak of 247.8 nm; **(D)** normalized by the Ge peak of 265.1 nm. Color images available online at www.liebertpub.com/ind

The areas under the Mg peak at 278.5 nm for all SRMs and non-SRM samples are plotted in *Figs. 2A* and *2B* as functions of estimated Mg concentration for GDs of 1 and 3 μs, respectively. The NIST-certified values were used as the best estimates of Mg concentration for the SRMs. (*Figs. S2* and *S3* compare the analytical results obtained from the three ICP-OES methods to the NIST-certified values for Ca, Mg, K, Na, Al, Fe, Mn and P. *Figs. S4* and *S5* present similar comparisons for the non-SRM samples.) Predictor variable Mg concentrations for the non-SRM samples were obtained by averaging the values obtained from the ICP-OES methods. In cases in which the results of one method disagreed significantly from those of the other methods, the average of results from the ICP-OES methods with the closest agreement were used to estimate Mg concentration. *Figures S4* and *S5* show the best estimates for concentrations of elements of interest, including Mg, in the non-SRM samples. The SRMs and non-SRM samples appear to follow similar trends and could be fit with a single calibration curve for Mg II 278.5 nm. However, this was not the case for all normalization techniques or elements analyzed. *Figures 2C* and *2D* are similar to *2A* and *2B*, except that the areas under the Mg peak have been normalized by the C peak at 247.8 nm and the Ge at 265.1 nm, respectively. Because the Ge and C peaks are approximately the same size for all samples, normalizing by C I 247.8 nm or Ge I 265.1 nm does not substantially alter the calibration fits, but only slightly improves some fits and worsens others. Normalizing by C I 247.8 nm or Ge I 265.1 nm does help in assuring that unexpected matrix effects and instrument drift do not substantially alter analytic predictions. Importantly, predicted Mg concentrations from Mg I 309.1 nm and Mg I 518.3 nm are in agreement with the data in *Fig. 2* (*Fig. S6*).

The *R*^2^ values and the 95% prediction uncertainties (*U*_95%_) for all of the displayed Mg fits are shown in *Table 2*. The *R*^2^ values for the SRMs are 0.97 or greater for all three Mg peaks for nearly all normalization methods, whereas fits to the non-SRM samples are much poorer for many cases. Importantly, however, normalizing the Mg peaks by C I 248 nm or Ge I 265 nm improves the fits for the non-SRM samples, raising their *R*^2^ values so they nearly match those of the fits for the SRMs. Interestingly, as noted in *Tables 1* and *2*, a GD of 1 μs provides the best calibration results for the non-SRM samples, while a GD of 3 μs provides slightly better fits for the SRMs, likely due to the fact that the lower Mg concentrations of the non-SRM samples benefit more from shorter GDs and larger detected intensities.

**Table 2.** Fit Parameters for Three Mg Peaks and Three Ca Peaks, Including Correlation Coefficients (*R*^2^) and Prediction Uncertainties (*U*_95%_) for GDs of 1 and 3 μs Without Normalization and Also Normalized by the C I 248 nm and Ge I 265 nm Peaks

		*R^2^*					*U_95%_*				
		GD = 1 μs			GD = 3 μs		GD = 1 μs			GD = 3 μs	
NORMALIZATION→		None	C248	Ge265	None	C248	None	C248	Ge265	None	C248
NIST SRMs	Mg 278.5	0.99	0.99	0.99	**0.99^a^**	0.99	342	498	469	**277**	413
	Mg 309.1	0.99	0.99	0.98	**0.99**	0.94	347	514	575	**335**	858
	Mg 518.4	0.98	0.97	0.98	**1.00**	0.97	544	719	553	**233**	647
Non-NIST SRMs	Mg 278.5	0.84	0.91	**0.99**	0.84	0.95	344	259	**83**	252	132
	Mg 309.1	0.40	0.43	**0.91**	0.48	0.68	668	650	**234**	450	351
	Mg 518.4	0.86	0.91	**1.00**	0.95	0.99	328	265	**51**	142	61
NIST SRMs	Ca 370.6	1.00	0.99	0.99	0.99	**1.00**	1144	1189	1409	1196	**809**
	Ca 849.8	1.00	1.00	0.97	0.99	**1.00**	1012	794	2484	1416	**691**
	Ca 315.9	0.98	0.99	0.98	0.99	**0.99**	2094	1898	2128	1582	**1098**
Non-NIST SRMs	Ca 370.6	0.89	**0.96**	0.84	0.82	0.88	157	**93**	176	191	158
	Ca 849.8	0.72	0.89	**0.90**	0.69	0.83	245	157	**144**	252	184
	Ca 315.9	0.68	**0.90**	0.88	0.28	0.45	265	**150**	154	381	334

^a^ Fit parameters for the best fits shown in bold type.

**Fig. 3.**
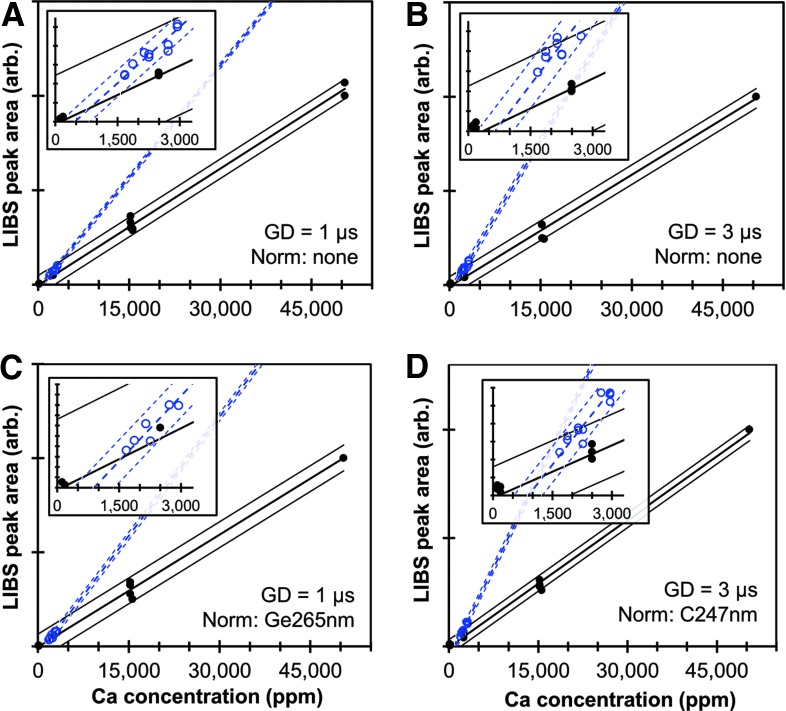
LIBS calibration data for calcium using the Ca II 370.6 nm peak. The area under the peak is plotted as a function of estimated Ca concentration for all SRMs (solid dots) and non-SRM samples (hollow circles). The thick solid and dashed lines represent the least-squares fit for the SRM and non-SRM datasets, respectively, while the thin solid and dashed lines represent the 95% confidence intervals for the SRM and non-SRM fits. **(A)** GD 1 μs; **(B)** GD 3 μs; **(C)** normalized by the C peak of 247.8 nm; **(D)** normalized by the Ge peak of 265.1 nm. Color images available online at www.liebertpub.com/ind

Following the format established in *Fig. 2*, the areas under the Ca peak at 370.6 nm for all SRMs and non-SRM samples are plotted in *Fig. 3* as functions of estimated Ca concentration for GDs of 1 and 3 μs and including normalization of the LIBS intensities by C I 247.8 nm and Ge I 265.1 nm. Importantly, the calibration curves for Ca are different from those for Mg in that the non-SRM samples appear to follow a distinctly different trend than the corresponding fits for the SRMs. Also, predicted Ca concentrations from Ca II 315.9 nm and Ca II 849.8 nm are in good agreement with the data in *Fig. 2* (*Fig. S7*). The *R*^2^ and *U*_95%_ values for all displayed Ca fits are also listed in *Table 2*. Again, the *R*^2^ values for the SRMs are all 0.97 or greater for all three Ca peaks for all normalization methods, whereas fits to the non-SRM samples are much poorer for many of the cases. Similar to Mg, normalizing the Ca peaks by C I 248 nm or Ge I 265 nm improves the fits, raising the *R*^2^ values to as high as 0.96, resulting in measurement uncertainties for Ca that are comparable to those for Mg. LIBS calibration curves for Al, Fe, K, Mn, Na, P, and Si are shown in *Figs. S8* through *S14*, and the fit parameters are provided in *Tables S1* and *S2*. Differences between SRM and non-SRM calibration curves are also observed for Fe and Mn and may also be present in fits for Al, K, Na, and Si.

## Discussion

There are several reasons why the non-SRM samples for some elements could follow a different calibration trend than that of the SRMs. The SRMs were prepared using a jet mill and then air separated to 75μ, while the non-SRM samples were ground to −80 μm using a knife mill. The differences in the milling procedures affect the particle size and shape distributions, which can affect the heat absorption and propagation from the LIBS laser pulse. The intense pelletization procedure at 700 MPa is intended to minimize the effects of the different milling procedures, but may not be completely effective. Other possible explanations for the differences in the calibration trends between the SRMs and the non-SRM samples are instrument drift, differences in moisture content of the samples, and errant calibration data. However, each of these possibilities is unlikely. The LIBS measurements were randomized with some repeated points and it was verified that equipment drift over the time duration of the experiments was not a significant factor and was corrected for by the peak normalization techniques employed. In previous experiments, moisture content up to 20% was also added to some samples prior to LIBS, but the moisture did not significantly affect the results (results not shown). Similarly, the calibration data from ICP-OES are not likely to be in error because consistent data were obtained from three separate methods and instruments at different laboratories. The differences between the calibration trends for the SRMs and the non-SRM samples highlights the requirement for LIBS analysis that new samples being analyzed closely match the calibration samples in terms of properties and preparation.

**Fig. 4.**
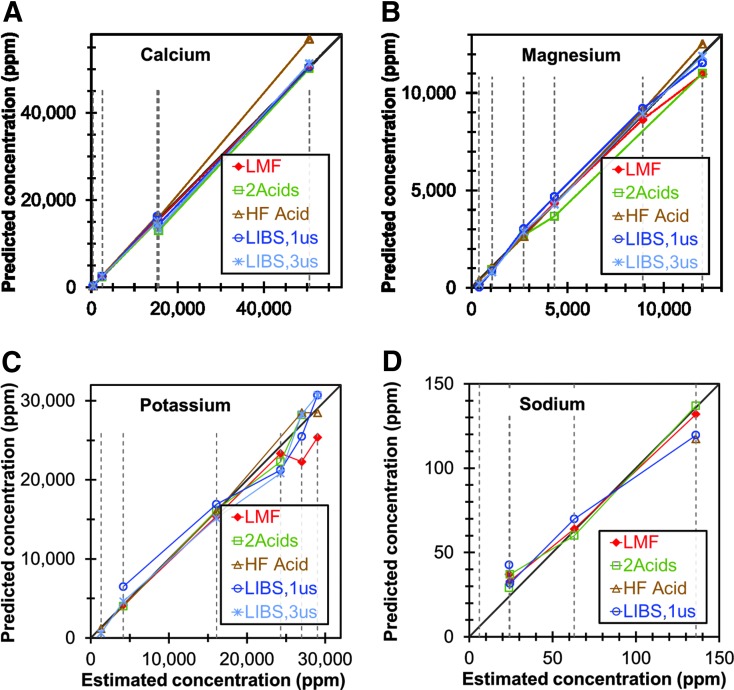
Comparison of concentrations of **(A)** Ca, **(B)** Mg, **(C)** K, and **(D)** Na of six SRMs predicted using LIBS (GD of 1 and 3 μs) with three ICP-OES/MS methods. All methods are plotted against their agreement to the NIST-certified values, shown by the dashed vertical lines. Color images available online at www.liebertpub.com/ind

The calibration results presented above demonstrate that LIBS is a promising rapid-screening technique for Al, Ca, Fe, Mg, Mn, P, K, Na, and Si using univariate models. Multivariate models were not employed in the present study because the number of samples in each distinct data set (six SRMs and six non-SRM samples) was not sufficient to assure that experimental noise did not adversely affect the reliability of the multivariate models. *Figure 4* plots the best available LIBS univariate calibration models and the results from the ICP-OES methods described above for the concentrations of Ca, Mg, K, and Na in the six SRMs as functions of the NIST certified values (*Fig. S15* shows a similar plot for Al, Fe, Mn, and P). For all of these elements, the LIBS method and all the ICP-OES methods yielded concentration values in good agreement with the NIST-certified values, with only a couple of exceptions, demonstrating that the LIBS method is comparable in accuracy and reliability to the three ICP-OES methods. Considering uncertainty in the NIST-certified values, additional errors due to sample splitting and equipment limitations, and the limited numbers of samples, it is difficult to determine which method is actually the most accurate.

**Fig. 5.**
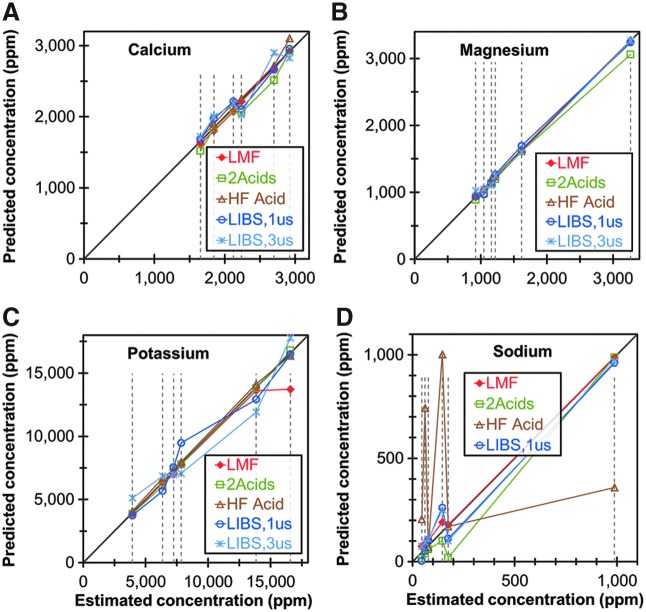
Comparison of concentrations of **(A)** Ca, **(B)** Mg, **(C)** K, and **(D)** Na for six non-SRM samples predicted using LIBS (GD of 1 and 3 μs) with three ICP-OES/MS methods. All methods are plotted against their agreement to the best estimated values, shown by the dashed vertical lines. Color images available online at www.liebertpub.com/ind

*Figure 5* compares the best-available LIBS univariate calibration models using GDs of 1 and 3 μs for the concentrations of Ca, Mg, K, and Na in the six non-SRM samples, with the concentrations of those elements determined using the three ICP-OES methods described above. (*Fig. S16* shows a similar plot for Al, Fe, Mn, P, and Si, including Si in the SRMs as measured by the ICP-OES methods).

The LIBS method appears to yield results that are comparable in accuracy and reliability to the ICP-OES methods for Al, Ca, Fe, K, Mg, Na, and Si. The LIBS method shows slightly greater disagreement from the ICP-OES methods for Mn and P. Of particular interest is the fact that the LIBS method appears to analyze Si more accurately in the non-SRM samples than it does in the SRMs, which is probably due to the relatively low concentrations of Si in the SRMs. It is also worth noting that the HF-acid method and the 2-acid method appear to be unreliable for Na and Al, respectively.

**Fig. 6.**
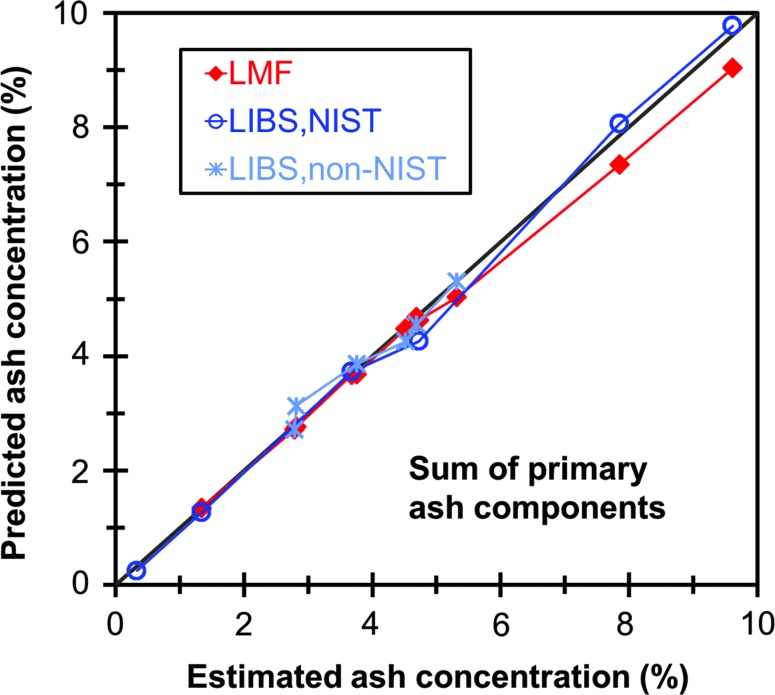
Concentrations of total ash (including Al, Ca, Fe, Mg, Mn, P, K, Na, and Si, excluding Cl and S) for SRMs and non-SRM samples as predicted by the lithium metaborate fusion (LMF) method and the best LIBS calibration curve for each constituent species. Estimated ash concentrations were determined by summing the contributions of the individual elements, using the NIST certified values for the SRMs and the estimated values for the non-SRM samples. Color images available online at www.liebertpub.com/ind

*Fig. 6* displays the sum of Al, Ca, Fe, Mg, Mn, P, K, Na, and Si concentrations—which comprise over 98% of the total ash for the non-SRM samples—for the SRMs and non-SRM samples as measured by the LMF method and the best LIBS calibrations. As shown, the LIBS method predicts total ash very well, and even better than the LMF method for samples with high potassium content, for which the LMF method may lose accuracy, as seen in *Fig. 5*.

Finally, it should be noted that the results presented here are valid for the LIBS instrument and methods used for this work. A different LIBS instrument with a different excitation laser, purge gas capability, collection optics, or detector could be expected to have somewhat different results. Other works have shown that reliable elemental analyses of samples prepared in the laboratory can be obtained using calibration curves from SRMs.^20^ The authors estimate that all of the necessary components to replicate these experiments, including a pulsed 1,064 nm laser, a 5-channel spectrometer system, automated two-dimensional sample stage, and other components could be purchased new for less than $30,000. Once the software was configured, such systems could be assembled with minimal effort, making LIBS a potentially low-cost method for rapid analysis of inorganic species and total inorganic concentrations in a wide range of biological and other materials. Despite the fact that the SRMs exhibited different calibration models than the non-SRM samples, for many biofuels applications in which a high level of accuracy is not required, the SRM calibration models could be used for non-SRM samples to estimate ranges of elemental concentrations and guide future processing decisions.

## Supplementary Material

Supplemental data

Supplemental data

Supplemental data

Supplemental data

Supplemental data

Supplemental data

Supplemental data

Supplemental data

Supplemental data

Supplemental data

Supplemental data

Supplemental data
