# Supplementary material for: Rapid Analysis of Inorganic Species in Herbaceous Materials Using Laser-Induced Breakdown Spectroscopy
Source: Ind Biotechnol (New Rochelle N Y). 2015 Dec 1;11(6):322–30. doi: 10.1089/ind.2015.0019 (PMC4693760; doi:10.1089/ind.2015.0019)
Supplement: Supplemental data [file Supp_Figure1.pdf]

Supplementary Data

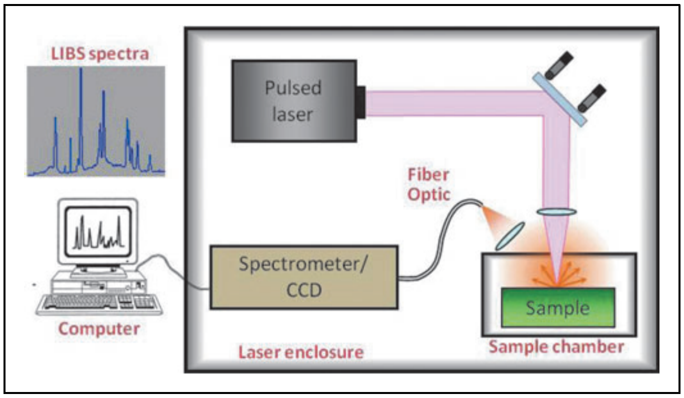

Supplementary Fig. S1. Schematic of the Model RT-100 LIBS setup (Applied Spectra, Fremont, CA).
