# Supplementary material for: Rapid Analysis of Inorganic Species in Herbaceous Materials Using Laser-Induced Breakdown Spectroscopy
Source: Ind Biotechnol (New Rochelle N Y). 2015 Dec 1;11(6):322–30. doi: 10.1089/ind.2015.0019 (PMC4693760; doi:10.1089/ind.2015.0019)
Supplement: Supplemental data [file Supp_Figure2.pdf]

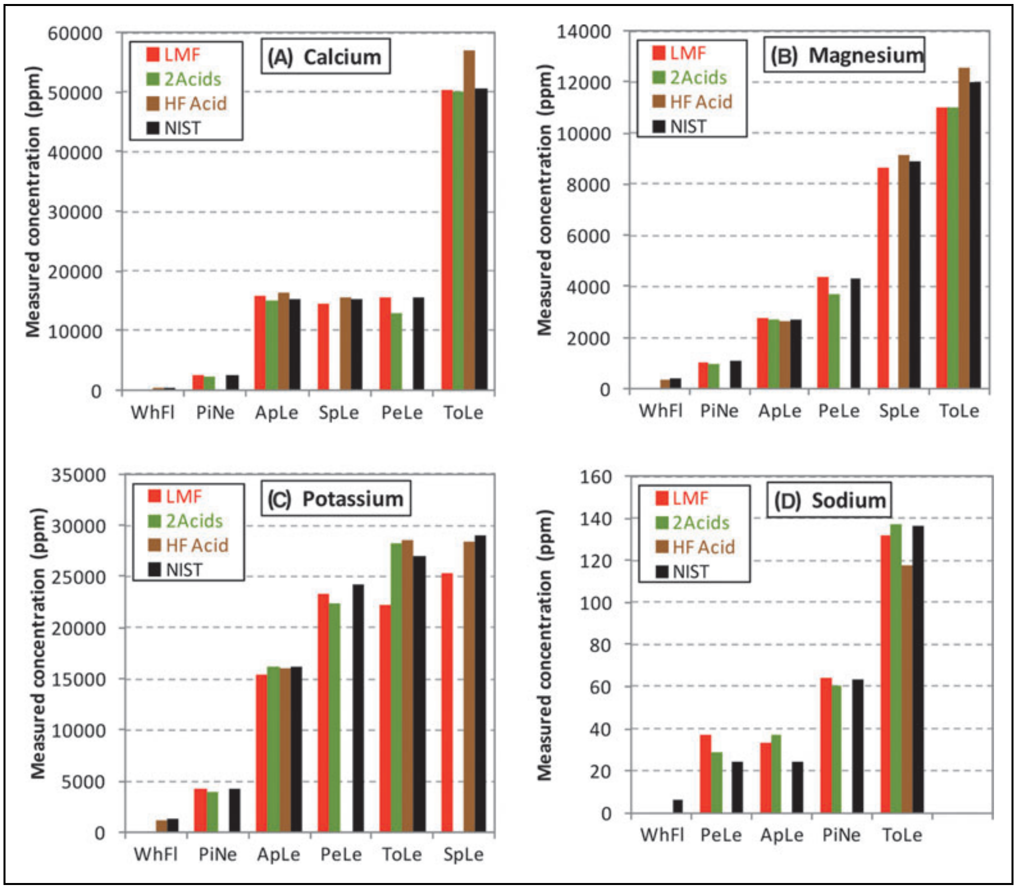

Supplementary Fig. S2. Measured concentrations of **(A)** Ca, **(B)** Mg, **(C)** K, and **(D)** Na for six NIST SRMs as determined using ICP-OES methods with HF-acid digestion, HNO_3- and $HClO_4$- acid digestions (labeled 2 acids), and a lithium metaborate fusion (LMF) method. *Figures S2* and *S3* compare the analytical results obtained from the three ICP-OES methods to the NIST-certified values for Ca, Mg, K, Na, Al, Fe, Mn, and P. For most elements, all three methods yielded results that are in reasonable agreement with the NIST values, although there are a few notable exceptions. Of particular concern are the reported values of Mg in tomato leaves and Na in peach leaves and apple leaves. In these examples, two of the test methods yielded similar values that were significantly different than the NIST-certified values, demonstrating that even in cases in which two independent methods yield similar results, there can still be significant error in the results.
