# Supplementary material for: Rapid Analysis of Inorganic Species in Herbaceous Materials Using Laser-Induced Breakdown Spectroscopy
Source: Ind Biotechnol (New Rochelle N Y). 2015 Dec 1;11(6):322–30. doi: 10.1089/ind.2015.0019 (PMC4693760; doi:10.1089/ind.2015.0019)
Supplement: Supplemental data [file Supp_Figure4.pdf]

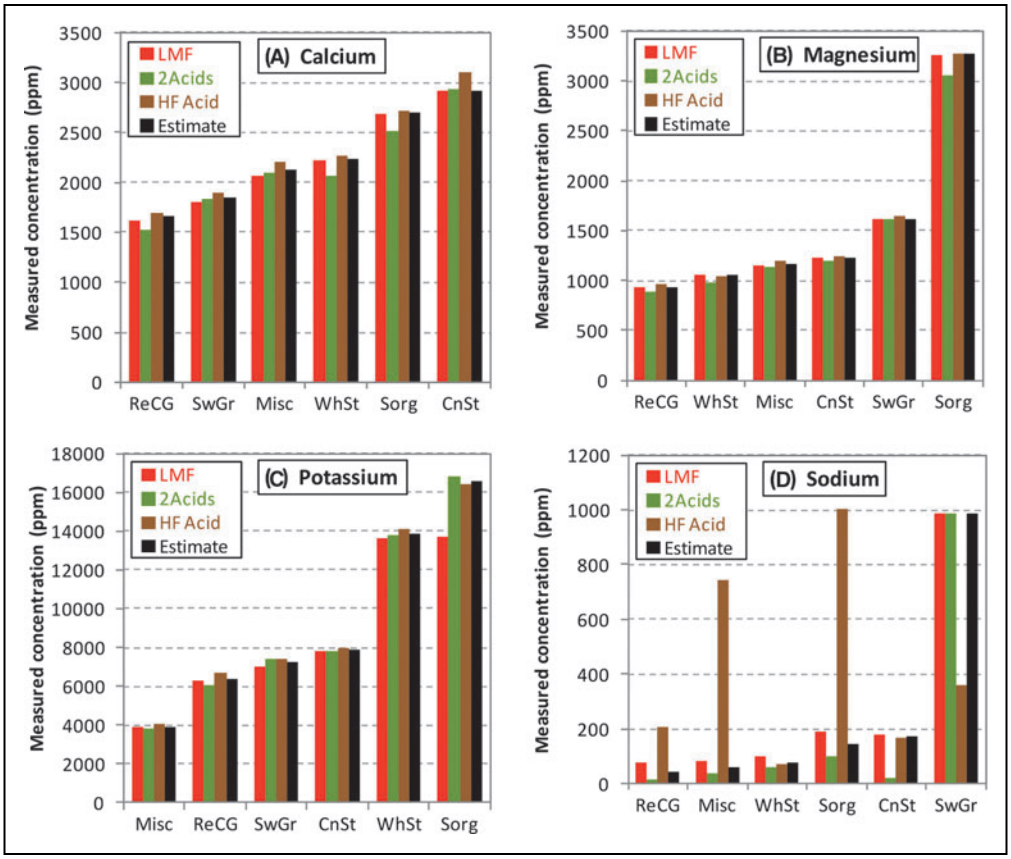

Supplementary Fig. S4. Measured concentrations **(A)** calcium, **(B)** magnesium, **(C)** potassium, and **(D)** sodium for six non-NIST samples as determined using ICP-OES methods with HF-acid digestion, HNO_3- and $HClO_4$- acid digestions (labeled 2 acids), and a lithium metaborate fusion (LMF) method. Elemental analyses from the three ICP-OES/MS methods are compared for the non-SRM samples in *Figures S4* and *S5*. The results are in good agreement for most elements for most materials; however, there are exceptions. Black vertical bars indicate the estimated concentrations for each element of interest (EOI) based upon the analytical results. The authors also note that due to limited time and budget, not all analyses were performed for all of the elements using all of the methods.
