# Supplementary material for: Rapid Analysis of Inorganic Species in Herbaceous Materials Using Laser-Induced Breakdown Spectroscopy
Source: Ind Biotechnol (New Rochelle N Y). 2015 Dec 1;11(6):322–30. doi: 10.1089/ind.2015.0019 (PMC4693760; doi:10.1089/ind.2015.0019)
Supplement: Supplemental data [file Supp_Figure5.pdf]

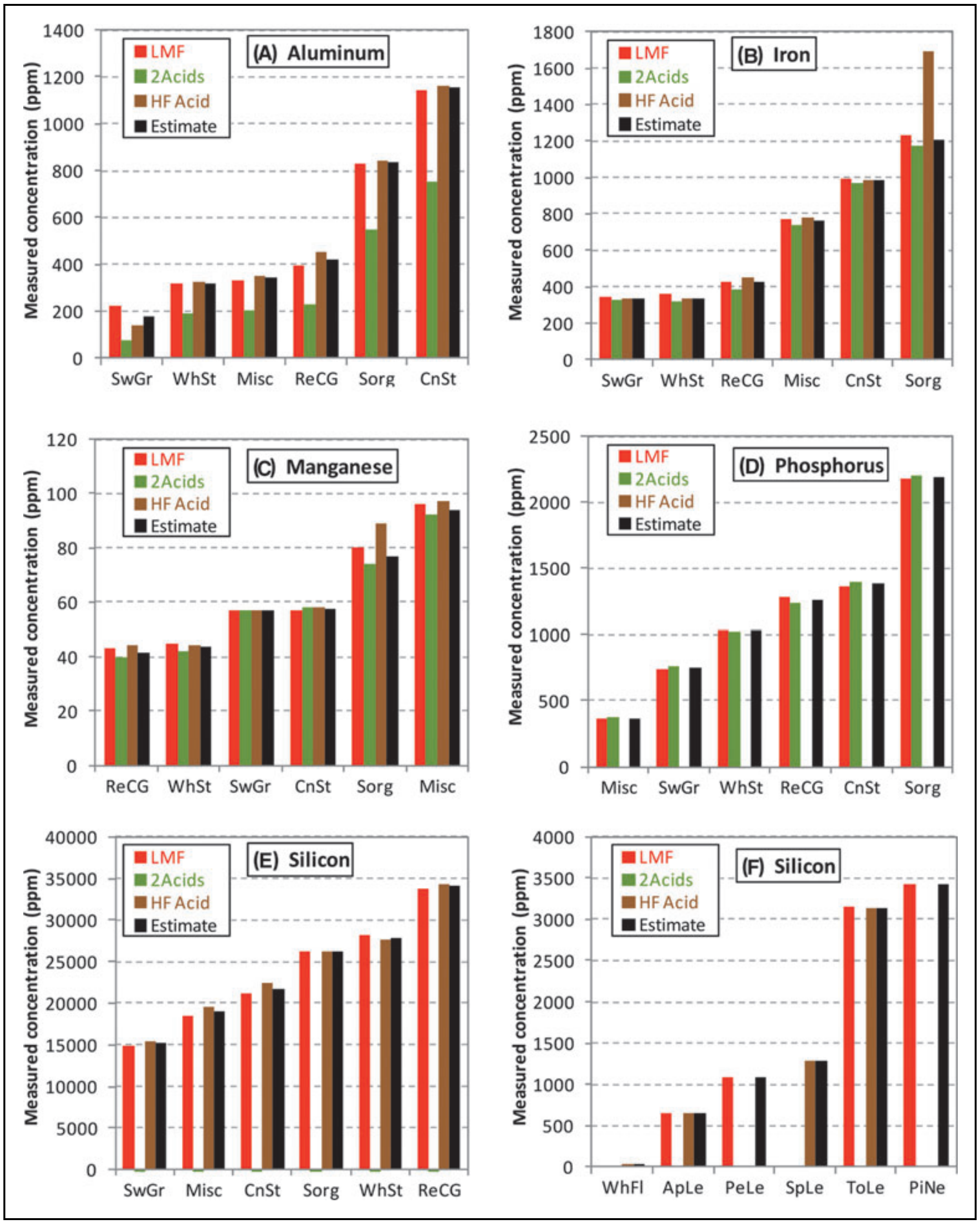

Supplementary Fig. S5. Measured concentrations **(A)** aluminum, **(B)** iron, **(C)** manganese, **(D)** phosphorus, and **(E)** silicon for six non-NIST samples as determined using ICP-OES methods with HF-acid digestion, HNO_3- and $HClO_4$- acid digestions (labeled 2 acids), and a lithium metaborate fusion (LMF) method; **(F)** contains similar Si concentration data for the NIST SRMs. For Al, the 2-acid method exhibited results that were significantly different than those of the other two methods for all samples. For the other elements, the results from the different methods were in fairly good agreement. Due to limitations, not all methods could be employed to analyze all elements. Elemental analyses from the three ICP-OES/MS methods are also compared for the non-SRM samples in *Figures S4* and *S5*. The results are in good agreement for most elements for most materials, however, as noted above, there are exceptions. Black vertical bars indicate the estimated concentrations for each element of interest (EOI) based upon the analytical results.
