# Supplementary material for: Rapid Analysis of Inorganic Species in Herbaceous Materials Using Laser-Induced Breakdown Spectroscopy
Source: Ind Biotechnol (New Rochelle N Y). 2015 Dec 1;11(6):322–30. doi: 10.1089/ind.2015.0019 (PMC4693760; doi:10.1089/ind.2015.0019)
Supplement: Supplemental data [file Supp_Figure6.pdf]

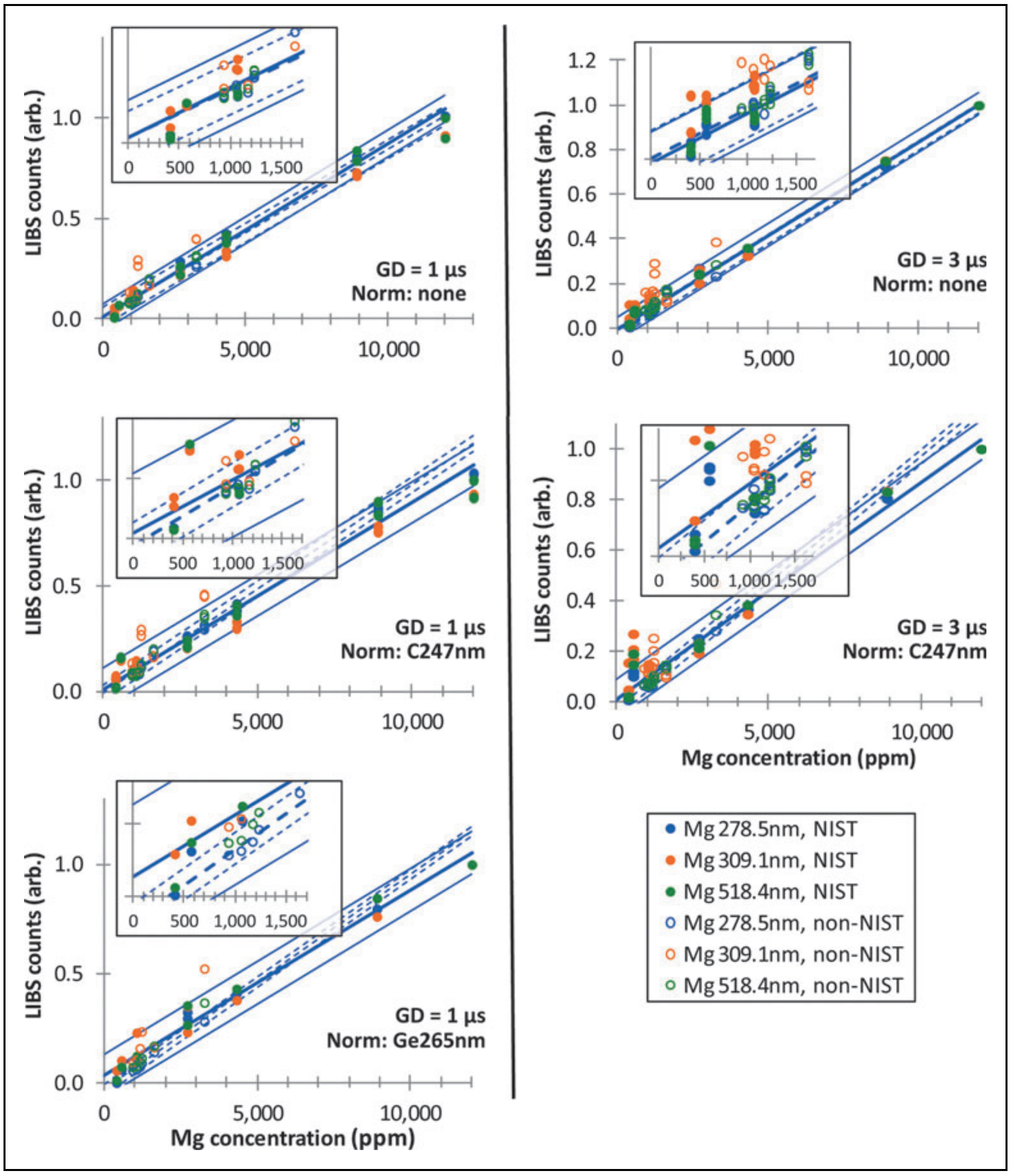


**Supplementary Fig. S6.** LIBS calibration data using Mg peaks at 278.5 nm, 309.1 nm, and 518.4 nm for GDs of 1 and 3 $\mu$s and including normalization of LIBS intensities by C I 247.8 nm and Ge I 265.1 nm; data from all three Mg peaks exhibit good agreement for nearly all samples.
