# Supplementary material for: Rapid Analysis of Inorganic Species in Herbaceous Materials Using Laser-Induced Breakdown Spectroscopy
Source: Ind Biotechnol (New Rochelle N Y). 2015 Dec 1;11(6):322–30. doi: 10.1089/ind.2015.0019 (PMC4693760; doi:10.1089/ind.2015.0019)
Supplement: Supplemental data [file Supp_Figure7.pdf]

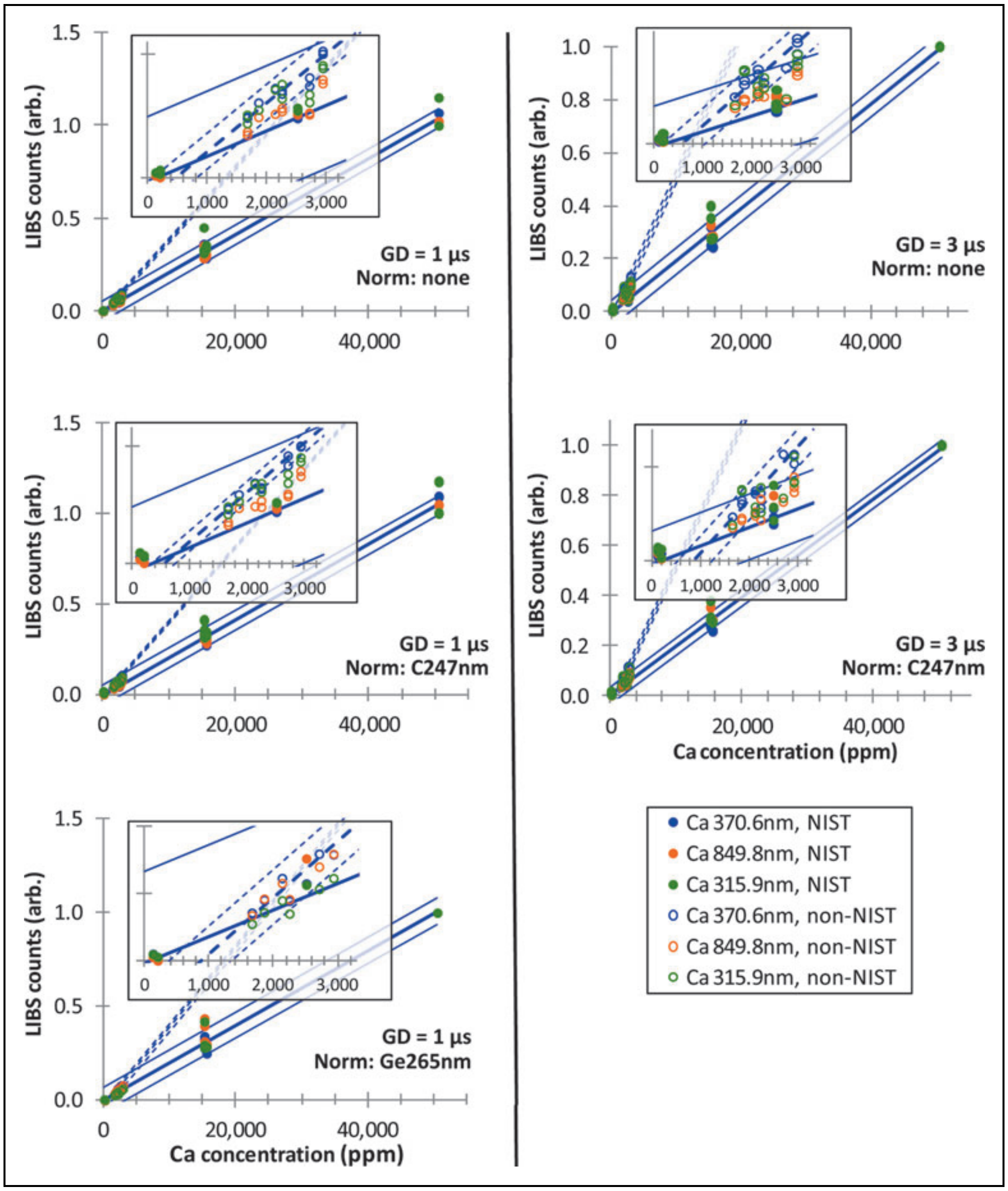

Supplementary Fig. S7. LIBS calibration data using Ca peaks at 370.6 nm, 849.8 nm, and 315.9 nm for GDs of 1 and 3 μs and including normalization of LIBS intensities by C I 247.8 nm and Ge I 265.1 nm; data from all three Ca peaks exhibit good agreement for nearly all samples.
