# Supplementary material for: Rapid Analysis of Inorganic Species in Herbaceous Materials Using Laser-Induced Breakdown Spectroscopy
Source: Ind Biotechnol (New Rochelle N Y). 2015 Dec 1;11(6):322–30. doi: 10.1089/ind.2015.0019 (PMC4693760; doi:10.1089/ind.2015.0019)
Supplement: Supplemental data [file Supp_Figure8-14.pdf]

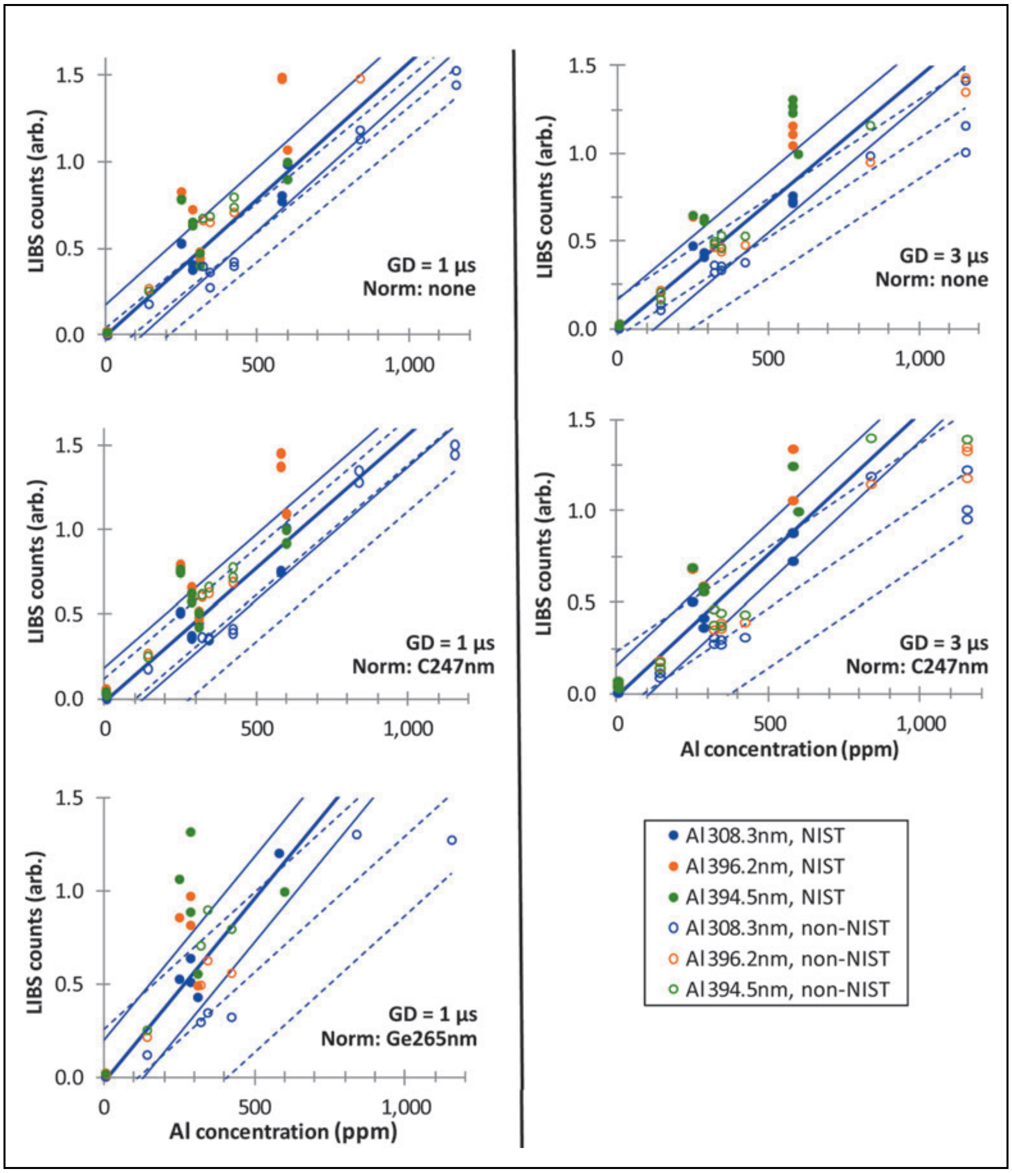

Supplementary Fig. S8. LIBS calibration data using the Al 308.2 nm, 396.2 nm, and 394.5 nm peaks and including normalization of LIBS intensities by C I 247.8 nm and Ge I 265.1 nm.

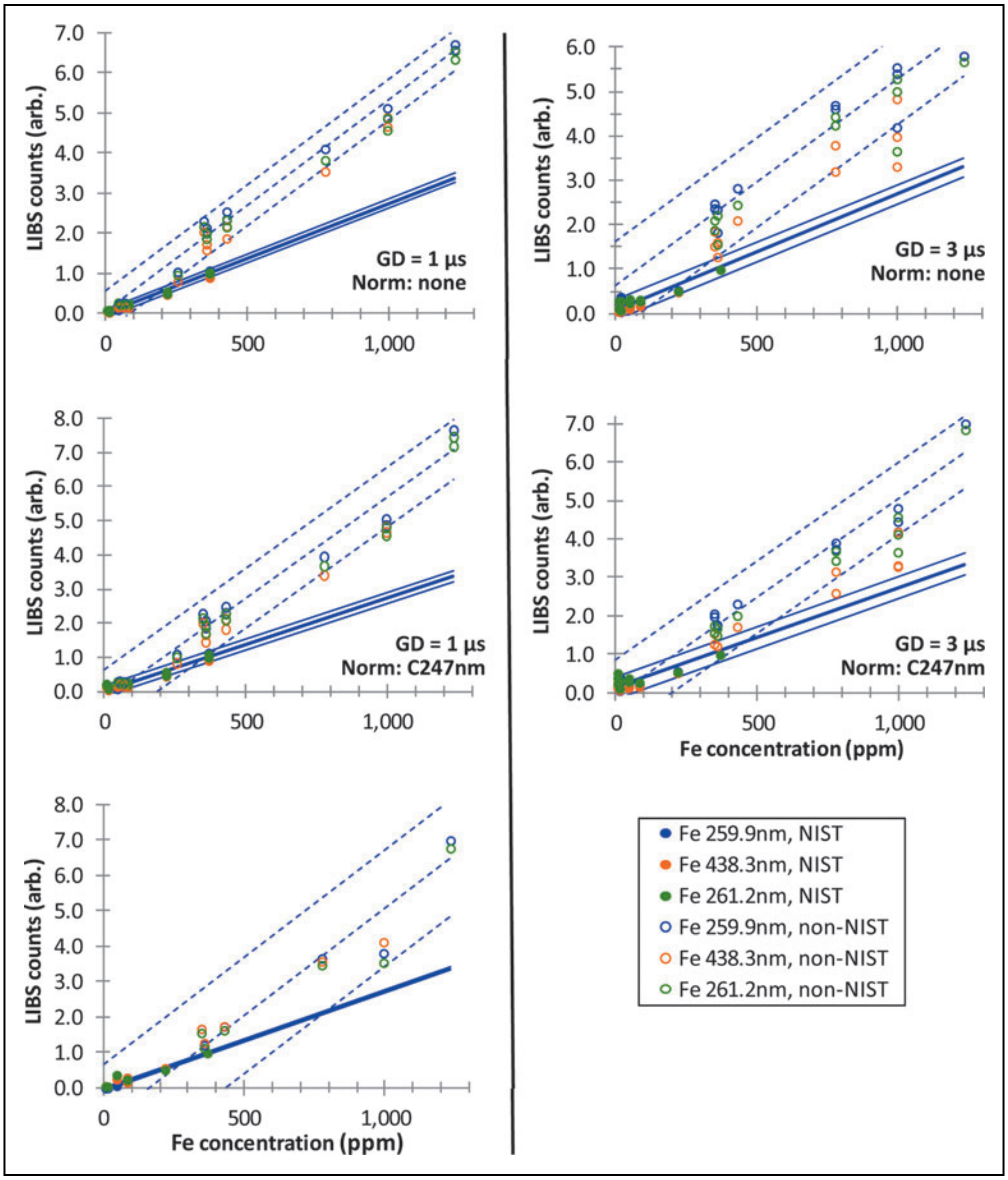

Supplementary Fig. S9. LIBS calibration data using the Fe 259.9 nm, 438.3 nm, and 261.2 nm peaks and including normalization of LIBS intensities by C I 247.8 and Ge I 265.1 nm.

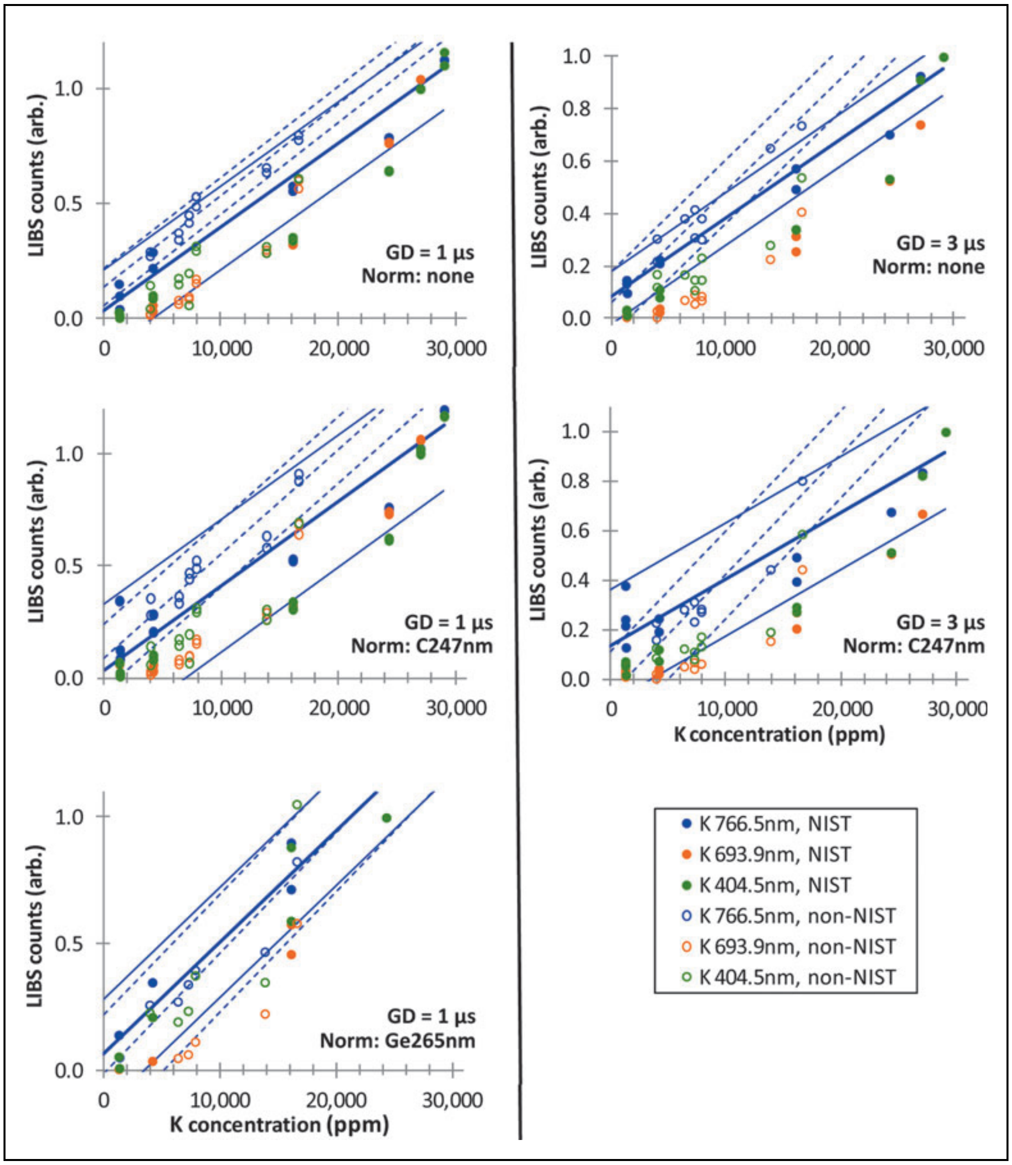

Supplementary Fig. S10. LIBS calibration data using the K 766.4 nm, 693.9 nm, and 404.5 nm peaks and including normalization of LIBS intensities by C I 247.8 nm and Fe I 265.1 nm.

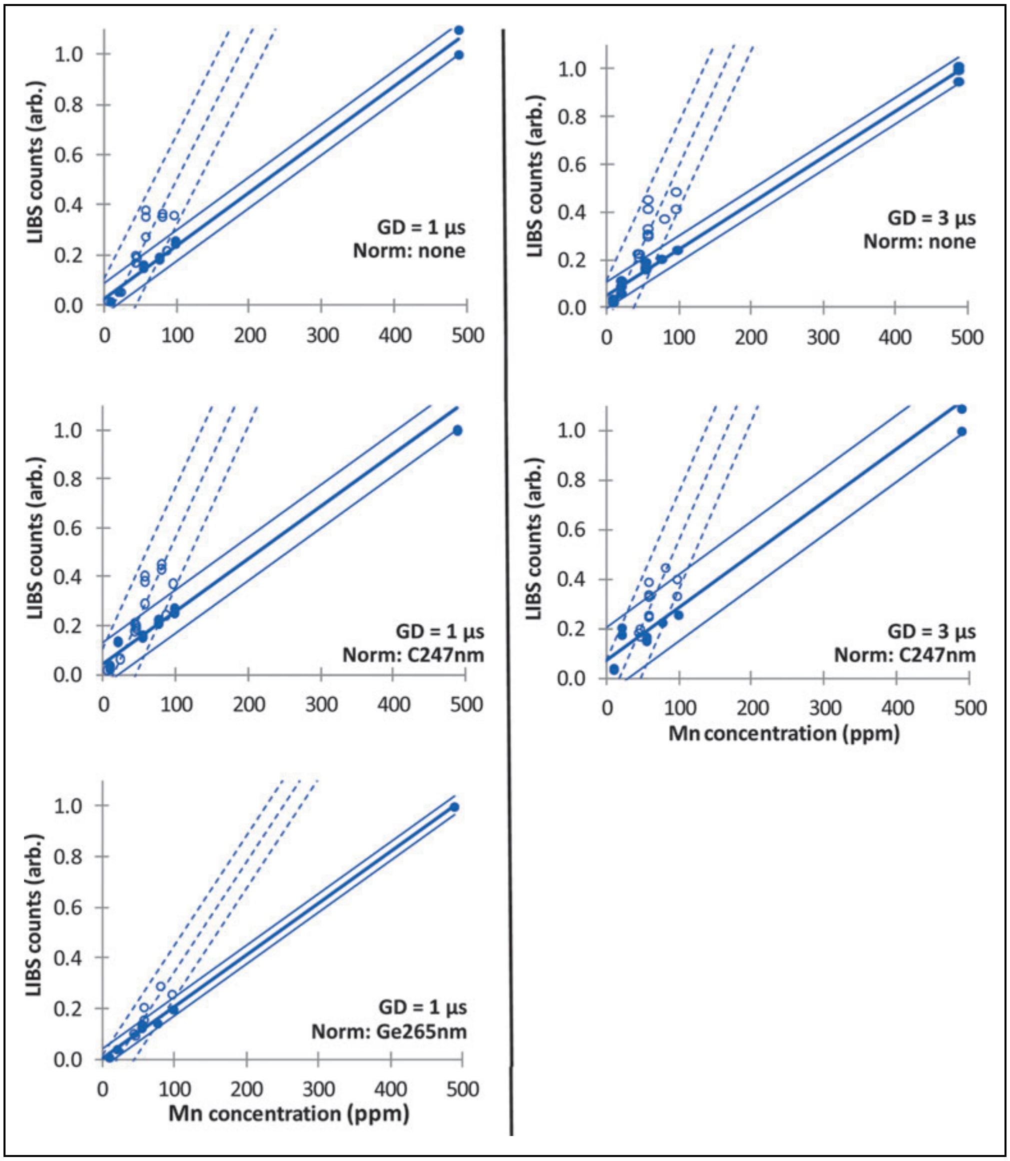

Supplementary Fig. S11. LIBS calibration data using the Mn 257.6 nm peak and including normalization of LIBS intensities by C I 247.8 nm and Ge I 265.1 nm.

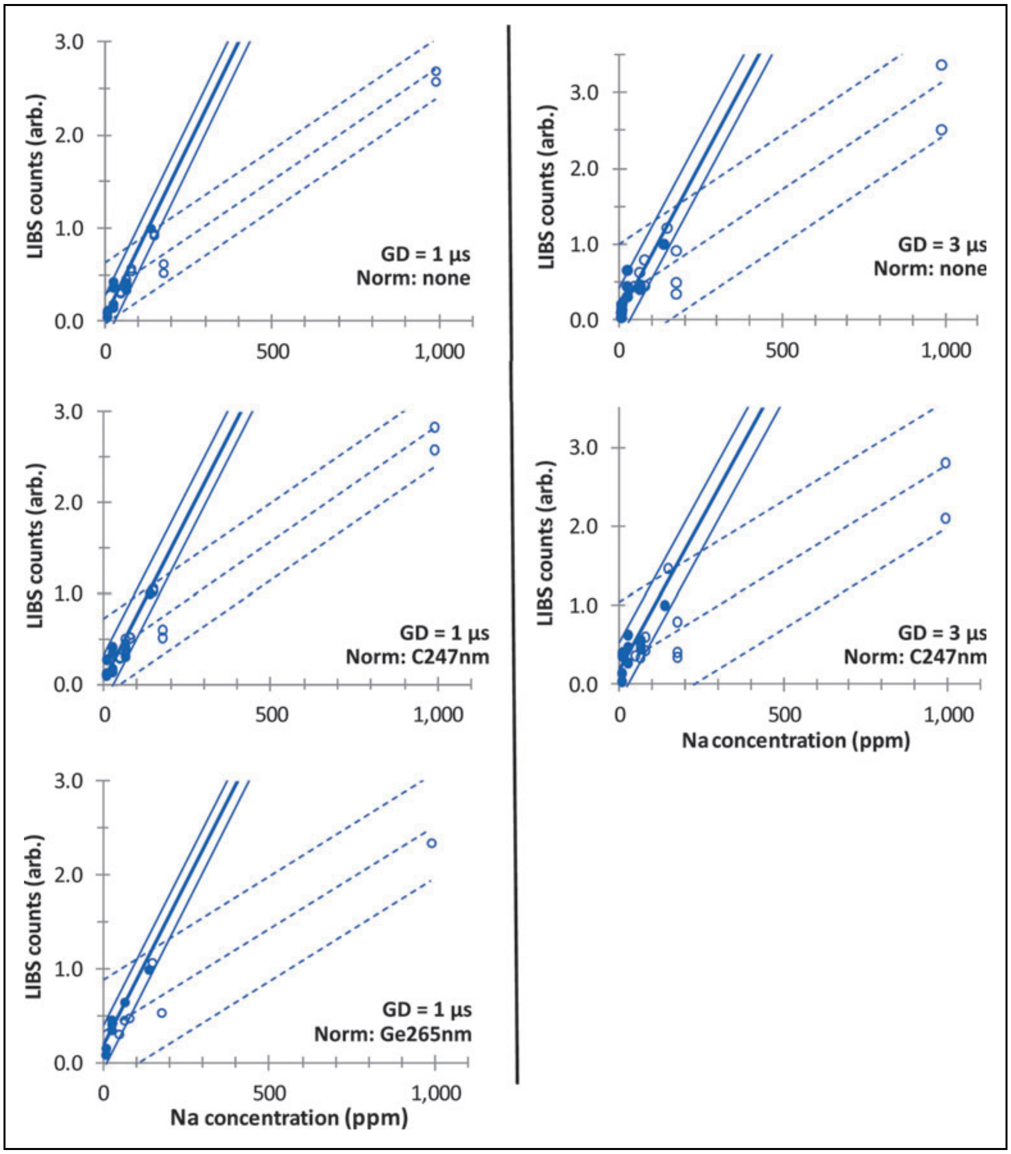

Supplementary Fig. S12. LIBS calibration data using the Na 588.9 nm peak and including normalization of LIBS intensities by C I 247.8 nm and Ge I 265.1 nm.

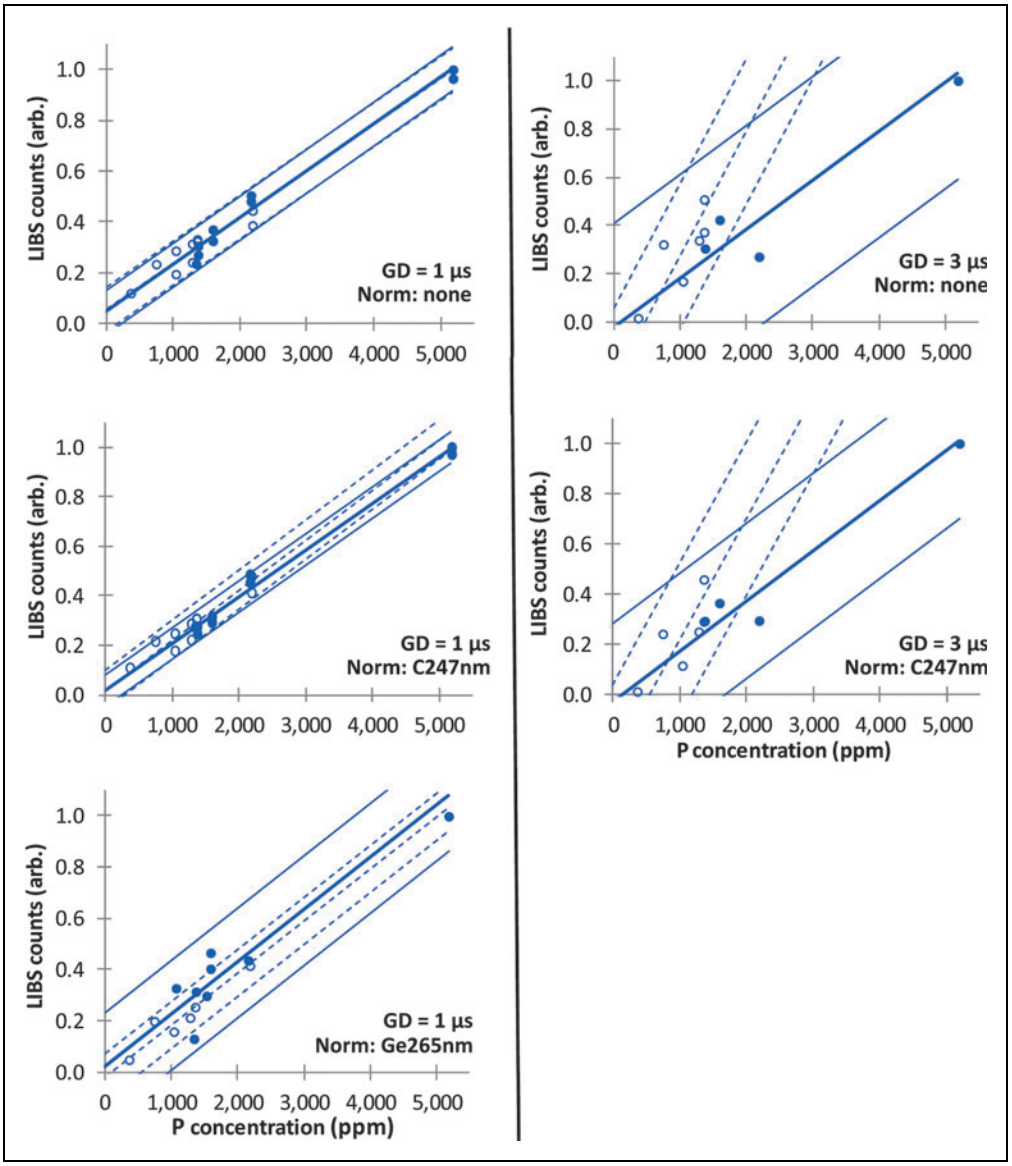

Supplementary Fig. S13. LIBS calibration data using the P 213.6 nm peak and including normalization of LIBS intensities by C I 247.8 nm and Ge I 265.1 nm.

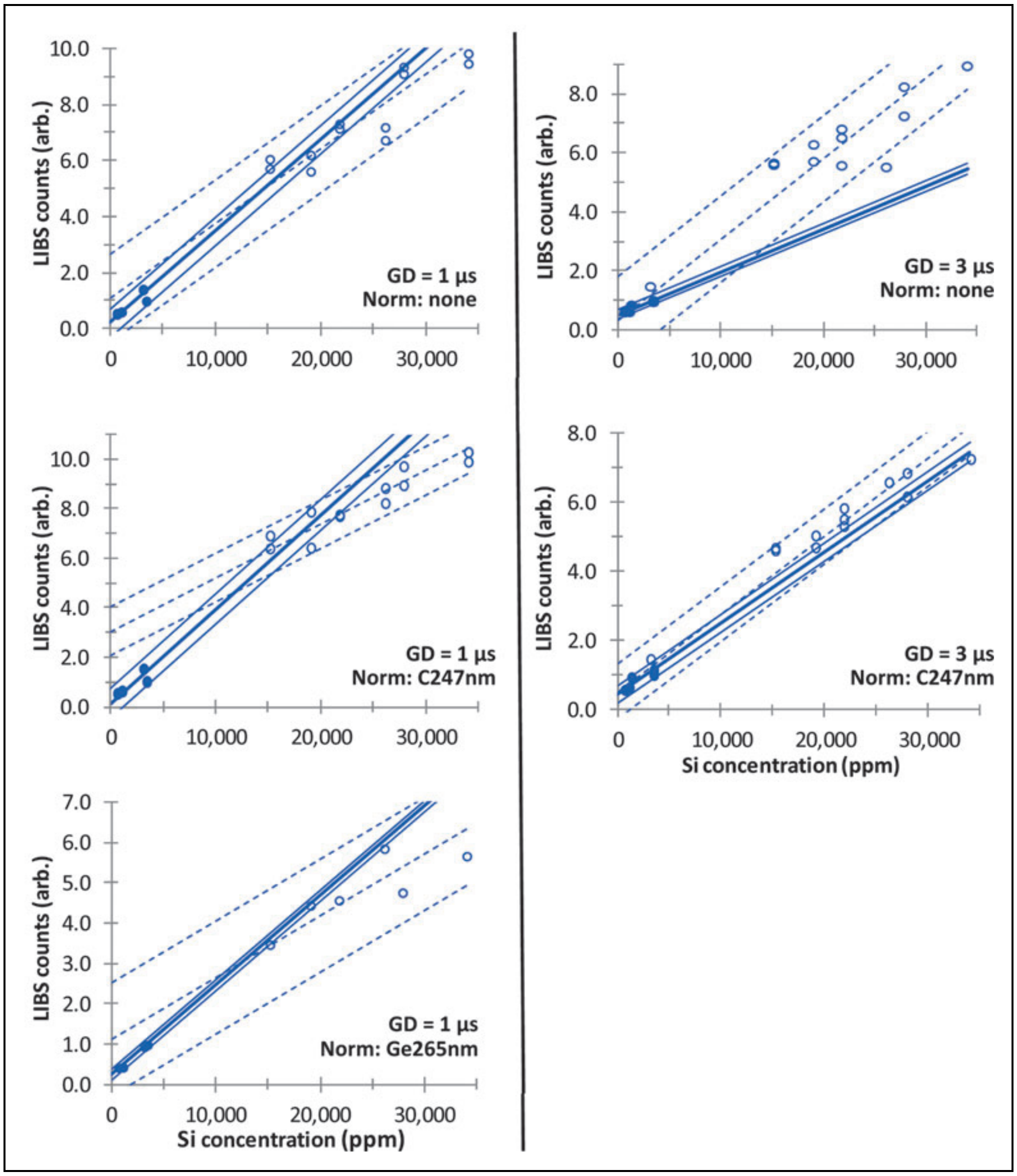

Supplementary Fig. S14. LIBS calibration data using the Si 251.6 nm peak and including normalization of LIBS intensities by C I 247.8 nm and Ge I 265.1 nm.
