# Supplementary material for: Rapid Analysis of Inorganic Species in Herbaceous Materials Using Laser-Induced Breakdown Spectroscopy
Source: Ind Biotechnol (New Rochelle N Y). 2015 Dec 1;11(6):322–30. doi: 10.1089/ind.2015.0019 (PMC4693760; doi:10.1089/ind.2015.0019)
Supplement: Supplemental data [file Supp_Table1.pdf]

Supplementary Table S1. Fit Parameters for Three Peaks Each of Al, Fe, K and Na, Including Correlation Coefficients (R^2) and Prediction Uncertainties ($U_{95\%}$) for GDs of 1 and 3 μs Without Normalization and Also Normalized by the C I 248 nm and the Ge I 265 nm Peaks

		R^2					$U_{95\%}$				
		GD = 1 μS			GD = 3 μS		GD = 1 μS			GD = 3 μS	
NORMALIZATION →		NONE	C248	GE265	NONE	C248	NONE	C248	GE265	NONE	C248
PEAK (NM) ↓											
NIST SRMs	Al 308.3	0.94	0.93	0.95	0.96	**0.97**[a]	52	56	48	51	**45**
	Al 396.2	0.84	0.87	0.70	**0.98**	0.95	86	77	114	**37**	54
	Al 394.5	0.75	0.78	0.55	**0.97**	0.94	107	100	138	**42**	64
Non-NIST samples	Al 308.3	**0.98**	0.95	0.91	0.95	0.89	**51**	78	106	88	128
	Al 396.2	0.90	0.88	0.95	**0.98**	0.97	112	119	80	**49**	72
	Al 394.5	0.91	0.89	0.94	**0.98**	0.96	106	116	82	**54**	76
NIST SRMs	Fe 259.9	0.98	0.96	**1.00**	0.87	0.79	17	27	**8**	37	48
	Fe 438.3	**0.98**	0.97	0.97	0.96	0.90	**18**	23	21	20	33
	Fe 261.2	**0.97**	0.95	0.92	0.90	0.72	**24**	29	34	33	55
Non-NIST samples	Fe 259.9	**0.99**	0.97	0.92	0.91	0.94	**44**	66	98	96	81
	Fe 438.3	**0.96**	0.92	0.86	0.86	0.75	**83**	115	129	118	160
	Fe 261.2	**0.99**	0.97	0.91	0.89	0.91	**46**	73	104	103	94
NIST SRMs	K 766.5	0.96	0.90	0.96	**0.98**	0.88	2260	3554	2014	**1488**	3735
	K 693.9	0.88	0.84	0.90	**0.92**	0.88	3934	4488	3376	**3034**	3676
	K 404.5	0.93	0.89	**0.94**	0.93	0.89	3059	3676	**2525**	2934	3553
Non-NIST samples	K 766.5	**0.96**	0.89	0.84	0.91	0.85	**912**	1444	1770	1210	1534
	K 693.9	0.91	0.86	0.86	**0.93**	0.81	1304	1660	1659	**1088**	1726
	K 404.5	0.77	0.73	0.67	**0.76**	0.68	2146	2280	2561	**1954**	2272
NIST SRMs	Na 589	0.86	0.82	**0.91**	0.73	0.67	14	16	**13**	19	22
	Na 819.5	0.71	**0.81**	0.39	0.71	0.77	21	**17**	33	20	18
Non-NIST samples	Na 589	**0.97**	0.95	0.92	0.90	0.83	**60**	75	92	110	140
	Na 819.5	0.52	0.36	0.92	**0.96**	0.94	232	268	92	**68**	85

[a]Fit parameters for the best fits are shown in bold type.
