# Supplementary material for: Rapid Analysis of Inorganic Species in Herbaceous Materials Using Laser-Induced Breakdown Spectroscopy
Source: Ind Biotechnol (New Rochelle N Y). 2015 Dec 1;11(6):322–30. doi: 10.1089/ind.2015.0019 (PMC4693760; doi:10.1089/ind.2015.0019)
Supplement: Supplemental data [file Supp_Table2.pdf]

Supplementary Table S2. Fit Parameters for Two Mn Peaks and Three Peaks Each of P and Si, Including Correlation Coefficients (R^2) and Prediction Uncertainties ($U_{95\%}$) for GDs of 1 and 3 μs Without Normalization and Also Normalized by the C 248 nm and the Ge 265 nm Peaks

		R^2					$U_{95\%}$				
		GD = 1 μS			GD = 3 μS		GD = 1 μS			GD = 3 μS	
NORMALIZATION →		NONE	C248	GE265	NONE	C248	NONE	C248	GE265	NONE	C248
PEAK (NM) ↓											
NIST SRMs	Mn 257.6	0.99	0.99	**1.00**[a]	1.00	0.98	13	18	**7**	13	28
	Mn 403.2	0.99	0.98	**1.00**	0.99	0.97	19	23	**7**	15	32
Non-NIST samples	Mn 257.6	0.67	0.67	**0.80**	0.57	0.52	14	14	**9**	12	13
	Mn 403.2	0.77	0.74	**0.74**	0.69	0.50	12	12	**10**	10	13
NIST SRMs	P 213.6	0.98	**0.99**	0.88	0.81	0.86	214	**170**	428	492	424
	P 255.3	0.95	**0.96**	0.89	0.87	0.84	314	**287**	408	407	452
Non-NIST samples	P 213.6	0.85	0.90	**0.92**	0.67	0.61	211	169	**162**	215	235
	P 255.3	0.77	0.87	**0.94**	0.63	0.68	260	191	**139**	228	212
NIST SRMs	Si 251.6	**0.75**	0.69	0.20	0.67	0.78	**604**	670	1146	766	624
	Si 243.6	**0.80**	0.74	0.19	0.70	0.76	**544**	623	1155	732	656
	Si 288.2	**0.87**	0.82	0.20	0.79	0.87	**441**	520	1143	616	489
Non-NIST samples	Si 251.6	0.82	0.89	0.71	0.95	**0.98**	2616	2047	3314	2563	**1702**
	Si 243.6	0.67	0.67	0.75	0.96	**0.99**	3523	3534	3080	2167	**1268**
	Si 288.2	0.78	0.89	0.84	0.95	**0.98**	2917	2086	2461	2501	**1368**

[a]Fit parameters for the best fits are shown in bold type.
