# Supplementary material for: Rapid Analysis of Inorganic Species in Herbaceous Materials Using Laser-Induced Breakdown Spectroscopy
Source: Ind Biotechnol (New Rochelle N Y). 2015 Dec 1;11(6):322–30. doi: 10.1089/ind.2015.0019 (PMC4693760; doi:10.1089/ind.2015.0019)
Supplement: Supplemental data [file Supp_Figure15.pdf]

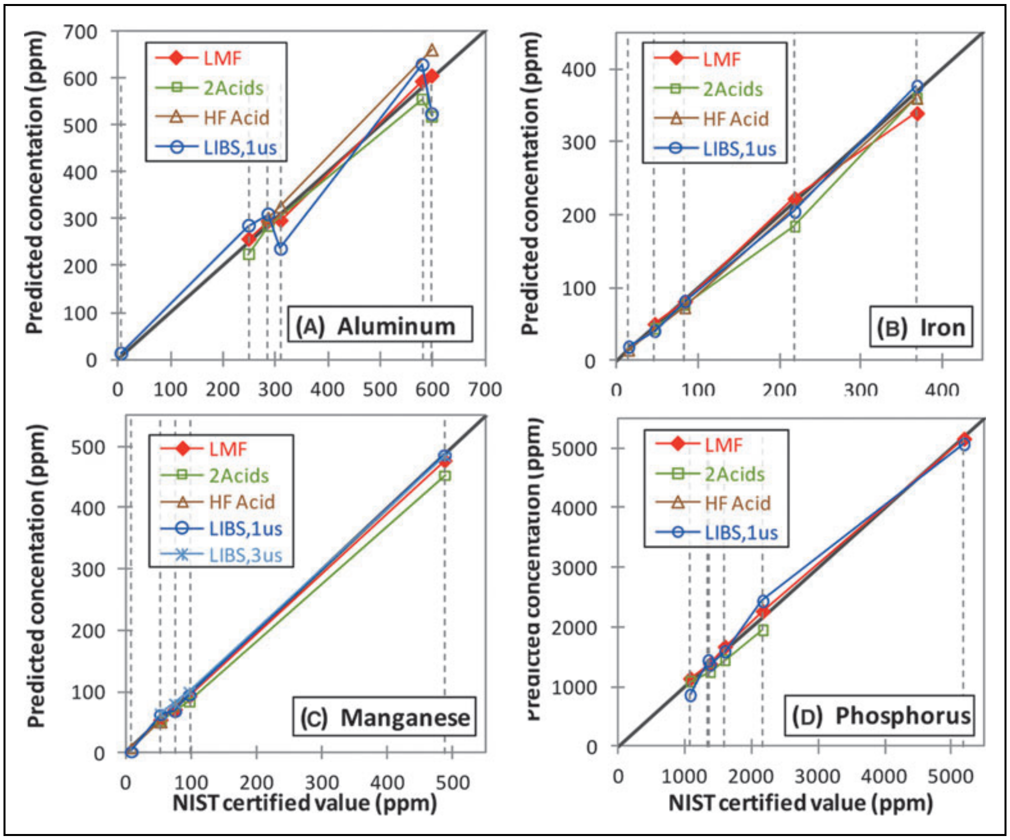

Supplementary Fig. S15. Comparison of concentrations of **(A)** Al, **(B)** Fe, **(C)** Mn, and **(D)** P of six SRMs predicted using LIBS (GDs of 1 and 3 μs) with three ICP-OES/MS methods. All methods are plotted against their agreement to the NIST-certified values, shown by dashed vertical lines.
