# Supplementary material for: Rapid Analysis of Inorganic Species in Herbaceous Materials Using Laser-Induced Breakdown Spectroscopy
Source: Ind Biotechnol (New Rochelle N Y). 2015 Dec 1;11(6):322–30. doi: 10.1089/ind.2015.0019 (PMC4693760; doi:10.1089/ind.2015.0019)
Supplement: Supplemental data [file Supp_Figure16.pdf]

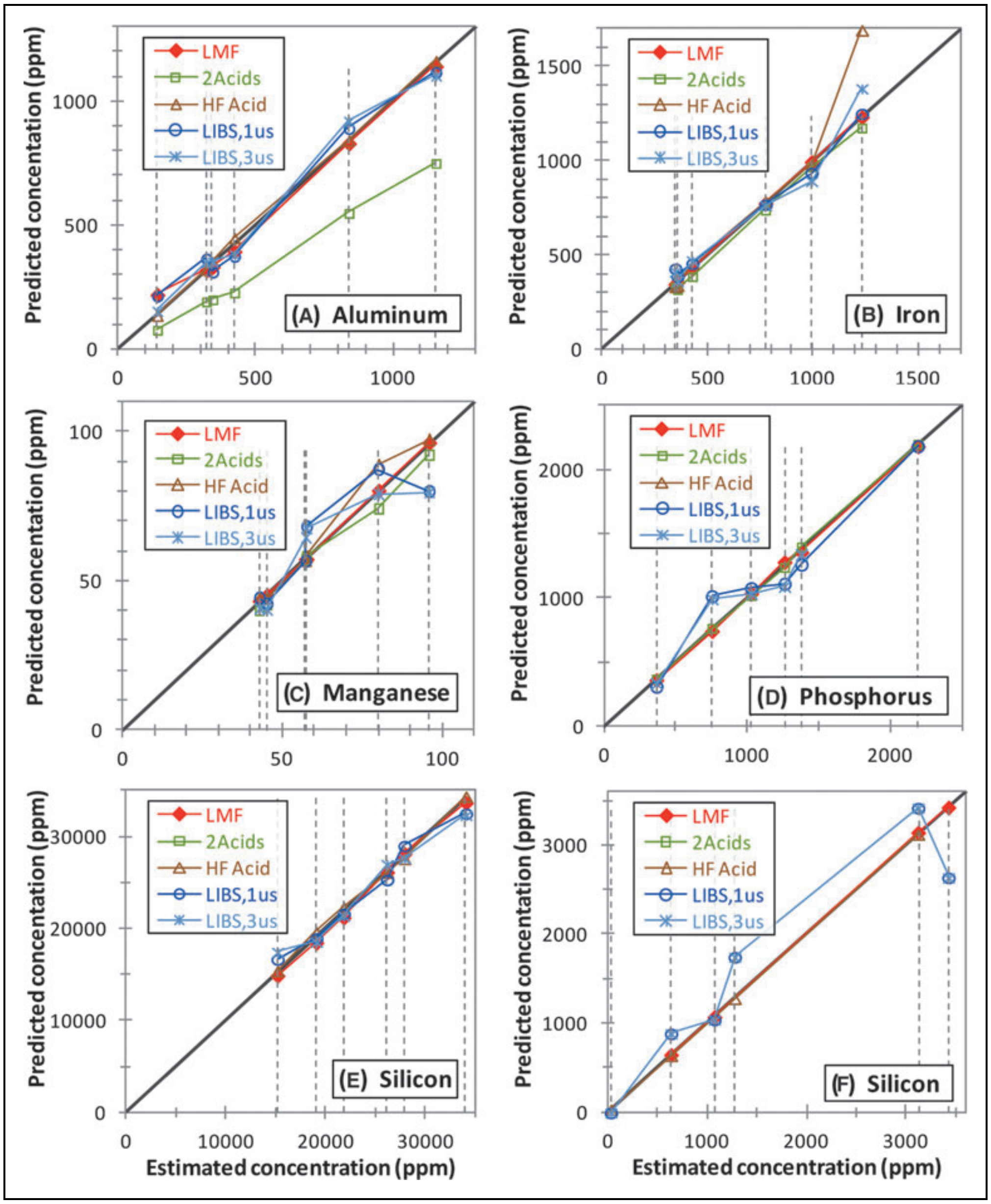

Supplementary Fig. S16. Measured concentrations of **(A)** Al, **(B)** Fe, **(C)** Mn, **(D)** P, and **(E)** Si for same non-NIST samples and using the same methods described in *Fig. 5*; **(F)** contains similar Si concentration data for the SRMs.
